# Linkage of Periodontitis and Rheumatoid Arthritis: Current Evidence and Potential Biological Interactions

**DOI:** 10.3390/ijms20184541

**Published:** 2019-09-13

**Authors:** Rafael Scaf de Molon, Carlos Rossa Jr., Rogier M. Thurlings, Joni Augusto Cirelli, Marije I. Koenders

**Affiliations:** 1 Department of Diagnosis and Surgery, School of Dentistry at Araraquara, Sao Paulo State University–UNESP, Araraquara 14801-903, Sao Paulo, Brazil; 2 Department of Rheumatology, Radboud University Medical Centre, 6500 HB Nijmegen, The Netherlands

**Keywords:** periodontal disease, rheumatoid arthritis, alveolar bone loss, bone, periodontitis, bone resorption

## Abstract

The association between rheumatoid arthritis (RA) and periodontal disease (PD) has been the focus of numerous investigations driven by their common pathological features. RA is an autoimmune disease characterized by chronic inflammation, the production of anti-citrullinated proteins antibodies (ACPA) leading to synovial joint inflammation and destruction. PD is a chronic inflammatory condition associated with a dysbiotic microbial biofilm affecting the supporting tissues around the teeth leading to the destruction of mineralized and non-mineralized connective tissues. Chronic inflammation associated with both RA and PD is similar in the predominant adaptive immune phenotype, in the imbalance between pro- and anti-inflammatory cytokines and in the role of smoking and genetic background as risk factors. Structural damage that occurs in consequence of chronic inflammation is the ultimate cause of loss of function and disability observed with the progression of RA and PD. Interestingly, the periodontal pathogen *Porphyromonas gingivalis* has been implicated in the generation of ACPA in RA patients, suggesting a direct biological intersection between PD and RA. However, more studies are warranted to confirm this link, elucidate potential mechanisms involved, and ascertain temporal associations between RA and PD. This review is mainly focused on recent clinical and translational research intends to discuss and provide an overview of the relationship between RA and PD, exploring the similarities in the immune-pathological aspects and the possible mechanisms linking the development and progression of both diseases. In addition, the current available treatments targeting both RA and PD were revised.

## 1. Introduction

**Figure 1:**
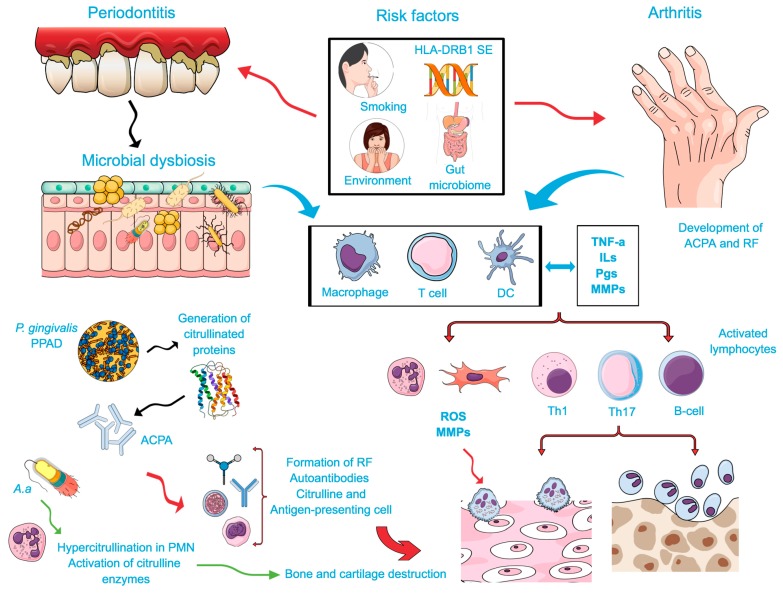
Possible biological intersections between rheumatoid arthritis (RA) and periodontal disease (PD): Common risk/predisposing factors and reciprocal biological influences. The exposure to certain environmental factors, e.g., smoking, genetic background (HLA-DRB1-SE), gut microbiome, infection with *P. gingivalis* and more recently with *A. actinomycetemcomitans* (microbial dysbiosis) leads to local protein alteration by citrullination. In combination with an inflammatory process stimulated by macrophages, dendritic cells, and T cells, a host response to citrullinated proteins in predisposed patients will occur. Immune cells will produce proinflammatory mediators (Interleukins (ILs), Prostaglandins (PGs), Tumor Necrosis Factor (TNF), and metalloproteinases (MMPs), which also contribute to the aggravation of the immune response. IL-17, an important cytokine of the Th17 induces the production of CXC chemokines, MMPs, and reactive oxygen species (ROS), as well as the osteoblast expression of the receptor activator of the factor nuclear kappa B ligand (RANK-L) that stimulate osteoclast activation. Stimulated lymphocytes (B and T cells, specifically Th1 and Th17) play an important function during bone resorption by means of the RANKL-dependent mechanism in both conditions. *P. gingivalis* infection lead to the activation of proteases and peptidylarginine deiminase (PPADs) that generates citrullinated proteins and triggers the synthesis of anti-citrullinated proteins antibodies (ACPAs). A resultant signal against citrullinated epitopes in the joints resulting in enhanced expression of the rheumatoid factor (RF) and ACPAs, assisting in the formation of immune complexes. *A. actinomycetemcomitans* lead to the hypercitrullination of neutrophils and result in the activation of citrulline enzymes, which are also involved in the breakdown of the immune tolerance to the host molecules. These immune complexes enhance the host inflammatory development, which may aggravate RA. In addition, the autoantibodies produced during this process might contribute to the inflammatory process by directly activating osteoclast and resulting in the bone and cartilage damage. Thus, citrullination may represent a biological mechanism bridging reciprocal influences between RA and PD.

The possible association between rheumatoid arthritis (RA) and periodontal disease (PD) has been investigated because of the numerous similarities in pathological and immunological characteristics, including: (1) Increased infiltration of inflammatory and immune cells including neutrophils, monocytes, and T and B lymphocytes; (2) increased release of pro-inflammatory mediators such as the tumor necrosis factor-α (TNF-α), interleukin-1β (IL-1β), interleukin-6 (IL-6), and matrix-degrading enzymes (MMPs, Cathepsin); (3) increased activation of the receptor activator of the factor nuclear kappa B (NF-κB) ligand (RANK-L) pathway induced by soluble mediators released by immune cells [1], with subsequent osteoclast differentiation and maturation. In addition, decreased levels of anti-inflammatory mediators, such as the IL-10 and transforming growth factor-β (TGF-β) are also reported in both RA and PD [2,3,4,5]. PD and RA also lead to systemic inflammation, indicated by increased levels of the C-reactive protein (CRP) in the plasma [6]. Environmental factors (smoking) and genetic background (gene polymorphisms) are also considered risk factors for both conditions. A summary of the overlapping features of RA and PD is presented in Figure 1.

In spite of differences in the etiologies of RA (autoimmune) and PD (dysbiotic microbial biofilm), there are similar biological processes involved, such as citrullination and autoantibody response [7,8] and the pivotal role of bacterial dysbiosis, which may represent direct links between these two conditions [1,9,10,11]. Citrullination of peptides is mediated by peptidylarginine deiminase (PAD) and is considered a key event in RA [12,13]. Recently, it was reported that the periodontal pathogen *P. gingivalis* express PAD, may represent a direct biological intersection between PD and RA [13,14,15,16,17]. Accordingly, recent studies have strengthened the hypothesis that PD is a risk factor for the RA development [18,19]. The authors showed that individuals at high risk to develop RA were presented with an increased prevalence of PD and periodontopathogenic bacteria (*P. gingivalis*) suggesting that PD is associated with disease initiation and could be targets for preventive interventions in RA.

In support to a biological link between PD and RA, there is a much higher prevalence of RA in PD patients (3.95%, compared to 1% in the general population) [20]. Interestingly, in the greater severity of RA (swollen joints), an increased erythrocyte sedimentation rate (ESR), and increased CRP are correlated with more severe periodontal bone resorption [21]. Previous case-control studies [22,23,24,25] showed that long-term smoking increases the risk for RA development. Furthermore, the epidemiological association between smoking and RA became the center of innumerous researches when smoking was recognized as a trigger to the citrullination of peptides via stimulation of the PAD enzyme [26]. In line of this, cigarette smoking has been associated with an increased risk of seropositive disease (ACPA and RF) [27]. Accordingly, a more aggressive form of PD, characterized by an increased periodontal attachment apparatus loss and bone resorption, is seen in smokers compared to non-smokers control patients [28,29,30,31]. However, it is important to bear in mind that several studies in the literature mention statistical or selection adjustment/control for smoking, and most of them (if not all) consider smoking as a categorical variable (current, former or never-smoker) and do not account for the frequency, type or duration of smoking. This is also true for the socioeconomic status of patients suffering from PD and/or RA. In this context, further randomized clinical trials are warranted to investigate the possible influence of cigarette consumption based on the frequency, type and duration of smoking in patients striking from both conditions.

This review discusses the information on mechanisms underlying the possible reciprocal influences between RA and PD, particularly those related to the similarities in the immune-pathological aspects, and also the current available therapeutic strategies targeting RA and PD.

## 2. Periodontal Disease

Periodontitis, the most common cause of tooth loss in humans [32], is one of the world’s most prevalent chronic inflammatory disease, affecting 46% of the United States population, according to the National Health and Nutrition Examination Survey (NHANES) [33], with 10–15% of the PD patients having a more aggressive form of the disease [34]. The ultimate outcome of PD is the loss/extraction of teeth that are no longer capable of supporting the functional demands, causing a significant impact on the oral health-related quality of life [35,36,37]. The economic burden of dental diseases is exceptionally elevated, with treatment expenses anticipated at 416 billion US dollars per year worldwide [38]. More than 70% of this spending goes toward the treatment of PD. In the United Kingdom, 3 to 4 million individuals (approximately 5% of the UK population) currently suffer from severe PD at a cost of 2 billion pounds/year to the National Health Service (NHS) [39]. In the past three decades, a number of studies has reported associations between periodontitis and various systemic inflammatory conditions, including arthritis, type 2 diabetes mellitus, and atherosclerosis [40,41,42].

PD is a chronic inflammatory condition of the supporting tissues (gingiva, periodontal ligament and alveolar bone) around the teeth, caused by a dysbiotic microbial biofilm on the tooth surfaces [34,43,44]. Most of the tissue destruction results from the host immune triggered and sustained by the dysbiotic process [34]. The dysbiosis causing the shift from the periodontal health to PD is analogous to that in the intestinal mucosal surfaces, as a stable microbial community comprised mostly by Gram-positive aerobes is changed to a pathogenic bacterial community characterized microaerophilic and anaerobic Gram-negative microorganisms. Three bacterial species are strongly correlated with PD: *P. gingivalis*, *Treponema denticola* and *Tannerella forsythia*, referred to as the “red complex” [45,46,47].

**Figure 2:**
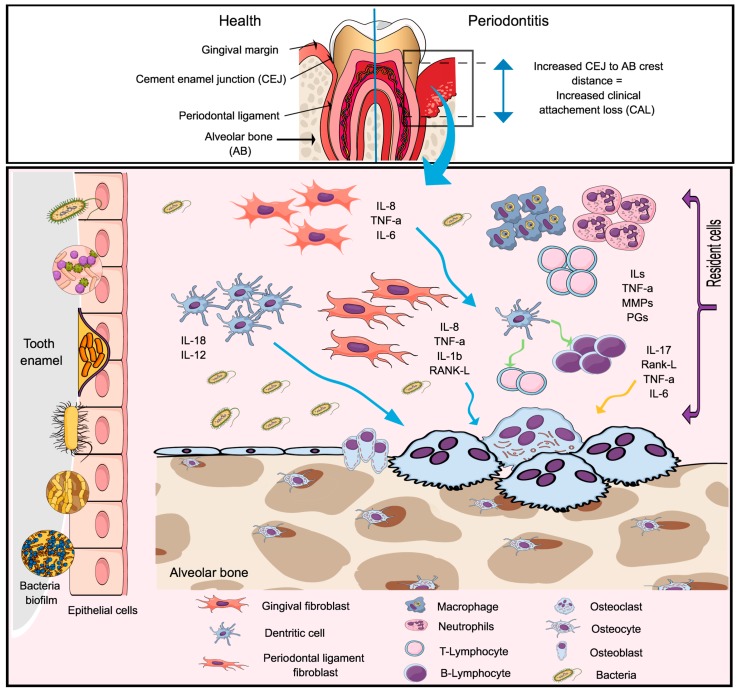
The pathogenesis of the periodontal disease. A dysbiotic microbiome localized in the enamel surface of the tooth, below the gingival margin, initiate the innate immunity by stimulating resident cells (epithelial cells, periodontal ligament fibroblast, and gingival fibroblast and dendritic cells) to produce mediators of inflammation in response to bacterial lipopolysaccharide (LPS) (via the toll-like receptor). Resident cells located in the connective tissue and alveolar bone produce proinflammatory cytokines and chemokines, including (Tumor Necrosis Factor- α (TNF-α), Interleukin-1 β (IL-1β), IL-6, IL-8, IL-12, IL-17 and the receptor activator of the factor nuclear kappa B ligand (RANK-L). Microorganisms located in the biofilm can reach the connective tissue and goes toward the alveolar bone, leading to the expression of RANK-L by osteoblasts, which can be accounted for the bone resorption seen during the disease process. If the infection fails to resolve, the release of pro-inflammatory mediators will continue and the activation of the B and T cells initiates the adaptive immunity. In this stage, the connective tissue become infiltrated by lymphocytes with predominantly more B cells (RANK-L) than T cells. The T cells will produce TNF-α, RANK-L and IL-17 which lead to increased osteoclastogenesis and bone resorption. This will result in the clinical signs of the disease characterized by increased clinical attachment loss (CAL.).

The molecular events that occur during PD pathogenesis are briefly described in Figure 2. Importantly, as a chronic inflammatory condition, both microbial- and host-derived molecular patterns (PAMPs and DAMPs) accumulate in the microenvironment alongside with the innate and adaptive immune cells [48,49,50] producing numerous inflammatory mediators, such as TNF-𝛼, IL-1𝛽, IL-6, IL-17, prostaglandin E2 (PGE2), growth factors, and degrading enzymes (collagenase, elastase, gelatinase), which are the main culprits of the destruction of the tooth-supporting structures [51,52,53], in a process somewhat analog to autoimmune conditions [54,55,56].

Variations in the host response to the microbial antigens derived from genetic influences and from acquired risk factors (e.g., systemic conditions affecting the immune response, medications, tobacco use) account for the significant variation in the susceptibility and severity of PD [2,57,58]. Like RA, PD is considered to be of a multifactorial condition, and systemic inflammatory disorders (such as RA) can lead to disturbing the equilibrium between the host and oral microbiota, assisting the onset of PD and/or enhancing the destruction of periodontal tissues.

Clinical characteristics of PD include friable gums, spontaneous bleeding, gingival recession, deepening pockets surrounding the tooth (indicating alveolar bone resorption), and eventual tooth loosening [59]. The diagnosis of PD is mainly based on clinical and intra-oral radiographic examination, involving bleeding on probing (BOP), probing pocket depth (PPD), clinical attachment level (CAL) and alveolar bone level [60]. The main therapy for the periodontitis treatment is based on the control of the oral biofilm by improving oral hygiene and removing plaque and dental biofilm through scaling and root planning. Of importance, there are variations in the periodontitis case definition and in the criteria for the severity of involvement that might compromise comparisons of the results reported by different studies. Moreover, few studies have reported the inflammatory status (bleeding on probing) as a criterium for the evaluation of the periodontal disease status.

## 3. Rheumatoid Arthritis

Rheumatoid arthritis (RA), a chronic autoimmune disease, is depicted by synovial inflammation and hyperplasia leading to irreversible damage of the cartilage and bone in the joints, loss of function, chronic pain and progressive disability (stiffness, swelling and deformation of the joints) [61,62,63]. RA affects up to 1% of the population worldwide, is three times more prevalent in women, and is associated with significant co-morbidities (cardiovascular illness, skeletal disorders such as: Periarticular bone loss, juxta-articular bone erosion, joint ankyloses and fractures) [5], socioeconomic burden, and mortality [4]. The exact etiology of RA is still poorly understood although it is hypothesized that the development of RA is dependent on the complex associations between environmental factors (e.g., long-term smoking), genetic background, hormonal, and infectious risk factors [64,65,66] resulting in the formation of autoantibodies and the onset of RA.

The diagnosis of RA is based on physical examination (according to the European League Against Rheumatism—EULAR criteria) [67], clinical history, and laboratory tests (presence of ACPAs and/or RF antibodies, abnormal acute phase reactants such as CRP and ESR), and also by imaging methods, such as magnetic resonance imaging (MRI) and ultrasonography methods [68]. The Disease Activity Score including the 28-joint count (DAS28) is also used in the clinical assessment of the disease activity calculated with a formula based on the number of swollen and tender joints, the patient’s global assessment based on a visual analog scale (VAS, 0–100) and measurements of CRP and ESR [69]. The management of RA is primarily based on medications, such as: Analgesics, non-steroidal anti-inflammatory drugs (NSAIDs), and biologic and non-biologic disease-modifying antirheumatic drugs (DMARDs), physical therapy, and surgery aiming at reducing symptoms, slowing joint damage progression and achieving remission [70,71].

**Figure 3:**
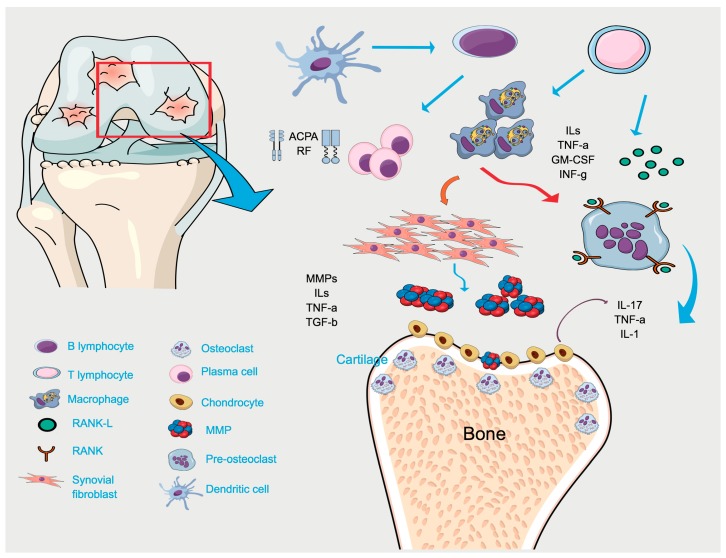
The pathogenesis of rheumatoid arthritis. The innate immune process is characterized by the infiltration of several inflammatory cells, chemokines, as well as other inflammatory mediators into the joint. Rheumatoid factor (RF) and anti-citrullinated proteins antibodies (ACPAs), the two most important autoantibodies, are typically produced by plasma cells. The coordinated production of proinflammatory cytokines and chemokines play crucial role in the orchestration of the inflammatory responses that ultimately result in the cartilage and bone destruction. Macrophages, synovial fibroblast, and dendritic cells produce several pro-inflammatory mediators such as Interleukins (IL-1, IL-2, IL-6, IL-10, IL-13, IL-15, IL-17, IL-18, Tumor Necrosis Factor- α (TNF-α), Granulocyte macrophage colony-stimulating factor (GM-CSF), and metalloproteinases (MMPs). In addition, the T cells activation leads to overproduction of inflammatory cytokines, including TNF-a, IL-1b, and IL-6 by macrophages. The overexpression of inflammatory cytokines enhances the capacity to induce the production of RANKL, which is the main regulator of osteoclastogenesis. These cells of the innate immune system hold extensive proinflammatory, destructive, and remodeling capacities, and substantially contribute to inflammation and joint destruction in RA.

The pathological aspects of RA include hyperplasia of the synovial membrane lining due to an influx of inflammatory cells (T and B lymphocytes, macrophages, neutrophils, and dendritic cells) into the synovium and joint cavity, resulting in the exacerbated production of cytokines and proteases. The overproduction of pro-inflammatory cytokines, such as TNF-α, IL-1β, IL-6, IL-17, the granulocyte macrophage colony-stimulating factor (GM-CSF), and RANKL is central to the pathogenesis of RA. These cytokines drive joint destruction by stimulating synovial fibroblasts and chondrocytes to secrete collagen-degrading enzymes (matrix metalloproteinases, MMPs) and by activating osteoclast differentiation leading to the cartilage and bone destruction [61,72,73,74,75]. The major cellular and molecular events in the pathogenesis of RA are represented in Figure 3.

In the last few years, there has been an increased focus on the role of citrullination and production of autoantibodies in the pathogenesis of RA. The level of anti-citrullinated protein antibodies (ACPA) is a highly specific marker of the disease (95–98% specificity) [59] and has been shown to be present in the serum of 70% of RA patients up to a decade prior to the initial effective diagnosis [2,76]. ACPA can be detected by a diagnostic test based on the reactivity against the synthetic cyclic citrullinated peptide (anti-CCP) [26,77]. The ACPA-positive RA is associated with a more severe disease compared to the ACPA-negative disease patients [78]. The presence of the rheumatoid factor (RF), a polyclonal antibody that reacts to the Fc portion of immunoglobulin G (IgG), is another diagnostic criterion for RA. In contrast to the ACPA, RF has only limited specificity for early detection of the disease and can be detected in several other diseases [79,80]. It has been shown that RF by itself does not contribute to the RA disease progression [81].

More recently, a role for the microbiome in the pathogenesis of inflammatory arthritis has been suggested as a possible modifier agent (environmental factor) in the disease development [82,83,84,85,86]. The connection of the gut microbiome in the arthritis pathogenesis was supported by the observation that experimental arthritis in both the IL-1 knockout and K/BxN animal models of RA was strongly attenuated under germ-free conditions [87,88]. Mucosal surfaces such as the lung, intestine and periodontal tissues are sites of the immune surveillance and a breach of the immune tolerance may contribute to development of arthritis [84,89]. This is sustained by the statement that autoantibodies (anti-PAD4, RF, antibodies against carbamylated proteins (anti-CarP), and ACPA,) might be distinguished years before individuals have joint symptoms [76,90]. Hypothetically, autoimmune responses initiated in microbially-colonized mucosal surfaces can transition to extramucosal sites like the synovial joints contributing to the signs and symptoms characteristic of RA [83,91,92,93]. Lately, it has been demonstrated that RA patients had a higher bacterial load, increased abundance of pathogenic species, and a more diverse oral microbiota associated with PD compared to the healthy controls (with RA and without PD), which resulted in worse periodontal condition (clinical attachment loss) in those patients. Moreover, changes in the oral microbiome (increased pathogenic species such as Prevotella, *Aggregatibacter actinomycetemcomitans* and *Parvimonas micra*) were associated with poor RA conditions (number of tender and swollen joints) in patients with RA-PD [43].

**Figure 4:**
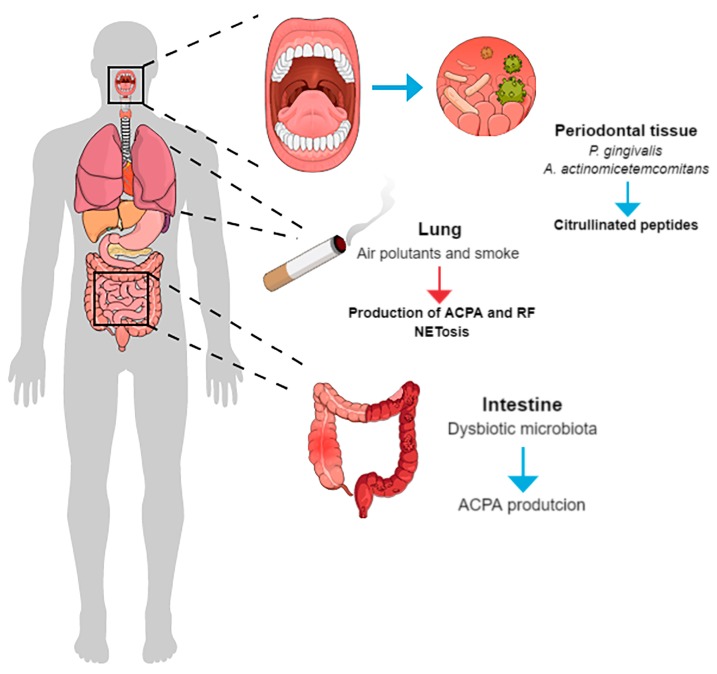
Potential initiators of immune-mediated inflammatory conditions at distant sites. A briefly description of the extra-articular potential initiators that might account for the pathogenesis of rheumatic diseases. Patients at high risk to develop autoimmune arthritis are more prone to infections due to endogenous (dysfunctional immune system) and external factors, i.e., periodontal disease and the presence of *P. gingivalis* and *A. actinomycetemcomitans* that trigger citrullinated peptides; exposure to risk factors such as smoke and pollutants might lead to the production of neutrophils extracellular traps (NEToses) and anti-citrullinated proteins antibodies (ACPA) in the lung; and the gut dysbiosis that also lead to the ACPA production. For patients at high risk to develop rheumatoid arthritis (RA), meticulous examining for infectious foci, particularly in the intestine and mouth, should be advocated in order to allow their early recognition and eradication.

In the context of the microbiome in the pathogenesis of rheumatic disease, microorganisms located in the gut and in the periodontal tissues (extra-articular) play an important role as potential initiators of immune-mediated inflammatory conditions at distant sites [94] (Figure 4). The oral environment possesses its own particular microbiota with more than 700 different species [95], which is located below the gingival margin (connective tissue) with a permeable epithelium. There are some hypotheses in which the microbiota may be involved in the progression of the disease, such as: Epithelial and mucosal permeability, loss of immune tolerance to components of the microbiota, and trafficking of immune cells to the joints [94]. In this context, periodontal pathogens can reach the blood circulation as a consequence of frequent and low intensity bacteremia induced by chewing or tooth brushing [96]. Once microorganisms gain access to the peripheral blood, they can colonize distant sites in the body and eventually initiate the pathological processes. Of importance, the DNA of *P. gingivalis, Treponema denticola, Prevotella intermedia, Prevotella nigrescens, Tannerella forsythia* and *Fusobacterium nucleatum* have been detected in the synovial fluid of patients with RA. Moreover, elevated titers of antibodies against *T. forsythia, P. intermedia* and *P. gingivalis* have been detected in the serum and synovial fluid of RA patients [97,98,99,100]. It has been suggested that an increased amount of Gram-negative microorganisms in the intestines increased toxic metabolites that reached blood circulation and may eventually enhance joint inflammation [101].

With significance to the pathogenesis of RA, there is a growing understanding of mucosal environmental exposures and dysbiosis as possible causal events during the onset of RA. One of the hypothesis raised is associated with the immunoglobulin A (IgA)-related autoimmunity (ACPA) as a potential causative link in the RA development, which is centered on the autoantibody isotype and plasmablast studies [102,103]. In this regard, Barra et al. [102] determined the prevalence of various ACPAs in first-degree relatives and found that the rate of the ACPA positivity in unaffected RA patients were very high. Moreover, in the first-degree relatives, ACPAs were not significantly associated with the SE, smoking, symptoms of RA, or PD. The ACPA profile of those patients consisted predominately of the IgA isotype and this outcome might be suggested as possible indicative of the IgA isotype in the pathogenesis of RA. Holers et al. elegantly confirmed that the lung also plays an important role in the pathogenesis of RA. They have demonstrated in subjects at high risk to RA development that the most citrulline-specific antibody response in the sputum were those to fibrinogen, vimentin, apolipoprotein E and fibronectin. Moreover, mucosal ACPA production in the lung, and possible in other tissues, is associated with the presence of local inflammation and increased levels of neutrophil extracellular traps (NET) formation. These findings supported the pivotal function of the lung in early RA related autoimmunity [104,105,106]. They also suggested that the most important initial event in the preclinical development of RA is the loss of the mucosal barrier function and systemic spread of an IgG ACPA response instead of the loss of tolerance to self-antigens [107]. However, studies evaluating mucosal origins hypothesis are limited because not all of them enable the relationship of the precise mucosal events present in the early time point or points during which such exposures would have influenced the concurrent autoimmune phenotypes as well as subsequent RA development. In this context, more studies are warranted to clarify those aspects.

For a comprehensive review on the role of mucosa-environment interactions in the pathogenesis of RA, the article made by Lucchino et al. [108] and a review paper made by the Holers group [107] are recommended.

## 4. Mechanistic Studies Linking RA and PD

Studies on the possible mechanisms by which both diseases may be interconnected explore the major molecular pathways associated with the pathogenesis of RA and PD.

### 4.1. Two-Hit Model Associating RA and PD

A possible association link between RA and PD is based on the theory of the “two-hit” model, first described by Golub et al. [109]. In this theory, the first “hit” involves the increased presence of anaerobic microorganisms and their antigens in the periodontal microenvironment. This initial hit triggers destructive events of periodontitis, such as increased production of bone-resorptive cytokines (IL-6, IL-1, TNF-α) and tissue-destructive proteinases (MMPs). The second “hit” involves a systemic disease, such as RA, that causes an increase of serological biomarkers of systemic inflammation (e.g., CRP, IL-6, IL-1β, PGE2, MMPs, and TNF-α). The increased serum levels of inflammatory mediators may further stimulate immune cells in the periodontium and enhance the production of MMPs and RANKL, aggravating destruction of non-mineralized and mineralized connective tissues in the periodontium [109,110], in a process that closely resembles cytokine-driven osteoclast activation and bone destruction during the pathogenesis of RA [26].

Similarly, Wegner et al. [111] suggested a “two-hit” model for PD influence on RA: The first hit is initiated by increased prevalence of PAD-producing *P. gingivalis* in the periodontal disease microenvironment, leading to increased local citrullination of peptidesband generation of APCAs. The second hit is represented by cross-reactivity of periodontal-generated APCAs to antigens present in the joint microenvironment, further aggravating the inflammation associated with RA. This amplification of the autoimmune reaction would end into the chronic and damaging inflammation that characterizes arthritis.

### 4.2. Genetic Susceptibility

Genetic variations are associated with both RA and PD. It is possible that some common genetic traits are associated with increased susceptibility to these conditions. One potential genetic influence connecting RA and PD is the shared epitope (SE)-coding HLA-DRB1 allele [112]. According to van der Woude et al. [113] 50% of the risk to develop RA is attributed to genetic factors, and the most relevant genetic association in RA is with the SE-coding HLA-DRB1 gene that confers more than 80% of susceptibility for joint destruction [64,114]. The HLA-DRB1 alleles encoding the beta chain of class II MHC can bind citrullinated peptides [115], possibly increasing the immunogenicity of the arthritis auto-antigenic citrullinated peptides [115,116]. It has been demonstrated that interaction between the elevated anti-RgpB IgG levels and the HLA–DRB1 SE was only detected in ACPA-positive RA patients [117], supporting a role for this genetic variation in the response to citrullinated antigens.

The SE-coding DRB1 alleles have been associated with bone erosions in RA as well as alveolar bone destruction during PD progression [118,119,120]. Recent data [112] shows that the transgenic mice carrying a human SE-coding HLA-DRB1 allele present spontaneous alveolar bone resorption and osteopenic skeletal changes, characterized by slenderer tibiae and decreased total bone area in the marrow and cortical tibial bones. Furthermore, overexpression of pro-inflammatory cytokines IL-17 and TNF-α were also found in SE-positive mice. SE acts as a signal transduction ligand that facilitates Th17 and osteoclast differentiation, increasing the RA severity [121,122,123]. These studies provided new insights in the association of SE with bone erosions in the inflammatory diseases supporting a genetic intersection between RA and PD.

The HLA-DRB1 SE has also been implicated as a risk factor for the periodontal disease [40,119,120]. Sandal et al. [124] demonstrated a potential role of HLA-DRB1 in the generation of ACPA in response to *P. gingivalis* oral infection model in mice. The generation of ACPAs in transgenic mouse (I-A°/I-E° on the C57BL/6 background that expresses susceptibility allele HLA-DRB1) required the expression of the PAD enzyme for the citrullination of host-derived proteins, representing a possible causative link between PD and RA [116]. This information supports the hypothesis of *P. gingivalis* as a possible source of the PAD enzyme inducing citrullination of host-derived peptides and the subsequent production of ACPAs that may cross-react with host-derived antigens in the joints and contribute/aggravate RA.

### 4.3. Bacterial Link Between PD and RA

Based on the microbial dysbiosis etiology of PD, increased interest has emerged in clarifying the potential role of specific bacterial species in the link with RA [125].

#### 4.3.1. The Citrullination Process

The indirect involvement of *P. gingivalis* in the pathogenesis of RA through the expression of PAD and the process of citrullination was first described in 2004 [45,111,126]. Citrullination is the process of post-translational modification of the amino acid arginine into citrulline, which is mediated by PAD, an enzyme of immune cells such as T and B lymphocytes, neutrophils, monocytes and macrophages [127], leading to the production of anti-CCP antibodies [15]. The isoform PADI4 is the most important for autoimmunity and is not active during homeostasis. When citrullinated proteins are formed in excess they can act as autoantigens, leading to the production of auto-antibodies favoring the pathogenesis of rheumatic diseases [128].

To date, *P. gingivalis* (the most common oral microorganism implicated in PD) is the only known microorganism with the ability to express the PPAD enzyme (known as PPAD to distinguish this bacterial enzyme from the human counterpart PAD). The PAD enzyme is directly associated with the formation of ACPA and plays an important role in the pathogenesis of RA [14,126]. The post-translational modification of arginine into citrulline through the PAD enzyme leads to the modification of the protein structure, and in genetically susceptible (e.g., in shared epitope-positive) individuals this might result in the generation of an immune response to citrullinated self-antigens. Citrullination associated with the host-derived PAD may be augmented by bacterial-derived PADs and enhance the production of ACPAs, which can precede the development of RA, and thus have an etiological role in its pathogenesis [129].

In translational studies, a comparison of wild-type (WT) *P. gingivalis* with PAD-deficient *P. gingivalis* or *P. intermedia* (without PAD) supports a role for PPAD as a mechanistic link between *P. gingivalis*-induced periodontal infection and RA [130]. WT *P. gingivalis* drastically augmented levels of autoantibodies to type II collagen and citrullinated epitopes whereas the PPAD-null mutant did not. Interestingly, administration of the protein arginine deiminase inhibitor (Pan-PAD inhibitor, Cl-amidine) diminishes the severity of the collagen-induced arthritis (CIA) in mice, indicating a causative connection between PPAD and RA [131].

In addition to its ability to express PPAD, *P. gingivalis* induces the production of pro-inflammatory cytokines (such as IL-6 and IL-1β) by the immune cells [132]. In this context, oral infection with *P. gingivalis* previous to the RA induction enhances the immune system stimulating a Th17 cell response that may accelerate arthritis development [133,134]. *P. gingivalis* has also the capacity to invade primary human chondrocytes when cultured in vitro, affecting cellular responses, which can contribute to the tissue damage during the RA pathogenesis [135,136]. All those characteristics of *P. gingivalis* suggest that PD, associated with increased prevalence of this microorganism, can influence the development of RA development through the citrullination process, the activation of Th17-related pathways. Taken together, this information supports a pivotal role for *P. gingivalis* in the causal link between PD and RA [137,138].

A clinical study investigated whether *P. gingivalis* influence the titer of ACPA in patients. They have concluded that in patients with PD, oral infection might be accountable for inducing autoimmune responses that characterizes RA [139]. It was suggested that RA-susceptible patients presenting PD may be exposed to citrullinated antigens produced by PPAD, which might lead to intra-articular inflammation [126]. PAD citrullinated peptides result in the expression of RF-containing immune complexes, leading to a local inflammatory reaction, both in the periodontal tissues and synovium, through Fc and C5a receptors [126]. This suggests a reciprocal influence of PD and RA, mediated by ACPAs and RF.

The prevalence of *P. gingivalis* in the oral microbiota of RA patients is strongly correlated with the presence of ACPA and it has been hypothesized that increased accumulation of citrullinated proteins and the reduced immunotolerance of RA subjects to citrullinated proteins lead to an increased formation of autoantibodies [140]. The increased formation of citrullinated proteins is associated with a more aggressive form of the disease and with earlier development of bone erosions [138]. A previous well-designed study has shown that ACPA concentrations were elevated among RA patients with PD and were associated with the presence of antibodies against *P. gingivalis*. The authors also showed that increased alveolar bone resorption was correlated with higher ACPA concentrations [137].

Although *P. gingivalis* is the PD-related microorganism most well-studied in the pathogenesis of RA, a recent study identified another periodontal pathogenic microorganism, *A. actinomycetemcomitans*, a Gram-negative coccobacillus, as a potential trigger for the pathogenesis of RA, providing a novel connection with PD [16]. This study established that *A. actinomycetemcomitans* induced hypercitrullination in host neutrophils by dysregulated activation of citrullinating enzymes through the pore-forming toxin leukotoxin A (LtxA—a major virulence factor of *A. actinomycetemcomitans*), resulting in a citrullinome that parallels as observed locally in RA-affected joints [16]. The same study also demonstrated that LtxA induced changes in the neutrophil morphology with the release of citrullinated proteins. Furthermore, exposure to leukotoxic *A. actinomycetemcomitans* was confirmed in RA patients with PD and was positively associated with ACPA levels. Emphasizing this relationship between the *A. actinomycetemcomitans* and RA development, the same group showed recently that the clinical symptoms of arthritis (morning stiffness, tenosynovitis, polyarthritis) and anti-CCP antibodies were successfully diminished when the antibiotic treatment against A. actinomycetemcomitans was prescribed for the patient, which was presented with *A. actinomycetemcomitans* endocarditis [141].

Furthermore, a potential role of the periodontopathogenic bacteria *P. intermedia* in the pathogenesis of RA was further elucidated in a study made by Schwenzer et al. [142]. This study was performed to clarify the mechanism by which PD could induce ACPAs, by examining the antibody response to a novel citrullinated peptide of cytokeratin 13 (CK-13) identified in the GCF of RA patients. The outcomes of this study have identified ACPA fine specificities associated with *P. intermedia.* Antibodies to cCK13-1 correlated strongly with anti-cTNC5, both of which were linked to a serologic response to *P. intermedia* infection pointing to the role of this bacteria in the pathogenesis of RA. However, previous pre-clinical studies [143,144] did not demonstrate the role of *P. intermedia* in the pathogenesis of RA, as described further below.

#### 4.3.2. Modulation of Immune Response by PD-Associated Bacteria

Oral inoculations with live *P. gingivalis* appeared to sensitize mice and rats to early RA development and increased the severity of joint destruction. An interesting study evaluated the primary role of *P. gingivalis* in the course of PD and subsequent RA in rats. The authors exposed the rats to oral inoculation with *P. gingivalis* and or *P. intermedia* for one month and then followed for eight months. PD was developed only in rats that received *P. gingivalis*. Interestingly, the RA development was confirmed in rats exposed only to *P. gingivalis* characterized by inflammatory infiltrate in the ankle joints with cortical erosions and cortical bone reduction. The authors concluded that oral priming with *P. gingivalis* triggered seropositive RA [143]. In line with this study, Maresz et al. have demonstrated that mice infected with *P. gingivalis* W83 strain developed antibodies to citrullinated peptides in a CIA arthritis model [130]. This means that infection with oral bacteria not only aggravates CIA but also seems to play a role in sensitizing mice to faster disease development and a more severe disease, with considerably more cartilage and bone damage in the joints. These outcomes were shown to be determined on the PPAD activity via increased inflammatory reaction and release of host PADs, leading to generation of citrullinated neo-epitopes. These findings were corroborated by another study showing that *P. gingivalis* aggravated the disease severity in an animal mouse model of RA and augmented the production of citrullinated antigens in the synovium [145].

Our group recently demonstrated a significant aggravation of arthritis severity in mice orally inoculated with *P. gingivalis* and *P. nigrescens* by increasing bone erosions in the affected joints [146]. These results indicate that the Th17 induction strongly depended on the TLR2 expression on antigen-presenting cells and was highly promoted by the IL-1 production, and stimulation of local Th17 cell differentiation. Moreover, this data provides evidence of the involvement of PD in the pathogenesis of the T cell-driven arthritis through induction of a Th17-type response [146]. We recently verified that oral infection with *P. gingivalis* aggravates AIA in mice, with aggravated bone erosion in the joints, associated with higher frequency of Th17 cells and increased levels of TNF-α and IL-17 [147]. Increased severity of AIA in this study is dependent of the Th17/IL-17 signaling pathway since the IL-17RA-deficient mice did not show bone loss or cytokine level alterations. The involvement of *P. gingivalis* in the activation of the immune system towards the Th17-pathways was further supported by increasing synovitis, bone erosions, and osteoclast numbers after PD induction with oral inoculation of live *P. gingivalis* [133]. For a complete overview in the role of Th17 cells in the pathogenesis of PD and RA, the review paper by Bunte and Beikler [148] is suggested.

Sato et al. [144] examined whether the modification of gut microbiota induced by *P. gingivalis* and *P. intermedia*, a Gram-negative microorganism also associated with PD that does not express PAD, is related with CIA. *P. gingivalis*, but not *P. intermedia* significantly aggravated CIA, with increased IL-17 levels in the serum, increased proportion of Th17 cells in lymphocytes, and a noteworthy change in the gut microbiome. Conversely, *P. gingivalis* did not increase the ACPA levels. These findings point to an exclusive role of *P. gingivalis* in the link between RA and PD by affecting the gut microbiota and inducing a shift to the Th17-type response.

A study using an oral inoculation model of the polymicrobial mixture of *P. gingivalis*, *T. denticola*, and *T. forsythia* to induce PD has shown that the presence of these bacterial strains exacerbate the development of CIA in mice [149]. Induction of PD in this study favored the CIA development, as shown by earlier commencement, and a more aggressive arthritic development associated with enhanced influx of inflammatory cells and pannus formation. Of importance, the identification of *P. gingivalis* in the joint tissues of mice with CIA challenged with periodontal pathogens suggests that a metastatic infection by PD-associated bacteria might contribute to the progression and severity of arthritis [149].

Bartold et al. [150] recognized that a combined approach using heat killed *P. gingivalis* to induce chronic inflammation followed by an arthritogenic hit to induce RA led to exacerbation of the immune response and increased severity of arthritis in rats. These findings were different from those described by Queiroz-Junior et al. [151] that showed no influence of *A. actinomycetemcomitans* induced-PD on the progression and severity of chronic AIA in mice. On the other hand, chronic AIA clearly exacerbated *A. actinomycetemcomitans* induced-PD. The lack of influence of PD on arthritis progression in this study [141] compared to the work made by Bartold et al. [150] might be attributed to differences in the bacterial species (*P. gingivalis* versus *A. actinomycetemcomitans*), the specific strain of bacteria used, route of bacterial infection (oral inoculation versus implantation of sponge impregnated with bacteria in the back), experimental animal species (mouse versus rat) and arthritis experimental model ( methylated BSA versus mycobacterium cell wall/adjuvant).

In line with these studies [150,151], Trombone et al. [58] reported that the association between bacterial induced-PD and pristane-induced RA in rats is dependent on a hyper-inflammatory phenotype. Interestingly, Queiroz-Junior et al. [152] have shown that AIA in mice provoked spontaneously inflammatory PD, without manipulation of the oral cavity. There is also evidence that pre-existing PD induced by oral inoculation with *P. gingivalis* exacerbated experimental arthritis using the CIA model in mice [153]. Intriguingly, mice with only PD showed evidence of bone resorption in the radiocarpal joint, and mice with only collagen-antibody induced arthritis demonstrated alveolar bone loss [153]. Taken together, these studies support the current hypothesis of a biological link between arthritis and bacterial induced-periodontitis.

## 5. Therapeutic Association in RA and PD: Epidemiological Studies

NHANES data indicates that the prevalence of PD, estimated by the number of missing teeth, is four times higher in RA patients [154]. This finding is corroborated by previous epidemiological and case-control studies, which showed that patients with active RA have significantly increased prevalence of PD (determined by different criteria, including bleeding, gingivitis, and increased probing pocket depth) compared to non-RA patients [137,155,156,157,158,159,160]. In counterpart, the prevalence of RA in PD patients is higher in comparison with matched periodontally healthy individuals [2,68,73,126,138,161,162,163,164]. Some studies [138,165,166,167] have shown that treatment of PD improves RA clinical/disease parameters (DAS28 score, CRP levels) and conversely that treatment of RA may decrease the extent of periodontal inflammation (Tables 1 and 2). This information strongly suggests that PD and RA are associated, and also that the nature of this association involves reciprocal biological influences.

### 5.1. Effects of Treating RA on PD

RA treatment involves various pharmacological approaches. The use of nonsteroidal anti-inflammatory drugs (NSAIDs—ibuprofen, aspirin and COX-2 inhibitors), glucocorticoids (GC) [168], and synthetic and biological disease-modifying antirheumatic drugs (DMARDs) such as, methotrexate, sulfasalazine, TNF-α inhibitors (infliximab, adalimumab, etanercept, certolizumab and golimumab) [169,170,171,172,173], IL-1β monoclonal antibody (canakinumab) [174], the IL-1 receptor antagonist (anakinra) [175], the humanized anti-IL-6 receptor antibody (tocilizumab) [176], and Janus Kinase (JAK) inhibitors (tofacitinib) [177,178,179,180] are currently the most prescribed drugs for the RA treatment. These classes of drugs reduce pain, inflammation and progression of the joint and therefore ameliorate the signs and symptoms of the disease, enhancing quality of life [114,181].

The long-term use of GC and NSAID by RA patients is associated with immune suppression, leading to oral changes such as xerostomia and candidiasis [182]. Although there is evidence from pre-clinical [183,184], and clinical studies [185,186], that drug-induced transient suppression of the immune response may attenuate PD, prolonged immunosuppression is also associated with a worse PD status in pre-clinical [187] and clinical studies [188,189]. This is reflected in RA patients, as some studies report worse periodontal status [73,155,190,191,192], whereas other studies demonstrate beneficial effects of the RA treatment (DMARDs) on the PD status. Table 1 summarizes the main outcomes of clinical trials evaluating the RA treatment in patients with established PD.

Most studies on the influence of the RA treatment on the severity of PD have focused on agents that target specific molecular factors in the inflammatory cascade, such as biological DMARDs. The TNF blockers used for the treatment of patients with RA resulted in significant reduction of biochemical markers of PD including IL-1β and IL-8 in the GCF of patients with established periodontitis [199]. Likewise, the anti-TNF-α treatment decreases periodontal indices and TNF-α levels in the GCF of patients with both autoimmune disease and periodontitis [200,202]. These studies suggested that the suppression of TNF-α to treat RA might also be beneficial in ameliorating PD. A systematic review with the meta-analysis [205] recently verified that the periodontal status of RA patients receiving antirheumatic medication was better than that of the untreated RA patients. These findings corroborate previous reports [197,198,206] showing a beneficial effect of adalimumab (a fully humanized monoclonal antibody), tocilizumab (a humanized monoclonal anti-human IL-6 receptor antibody), and rituximab (anti-B lymphocyte) therapy on the clinical periodontal conditions, as evidenced by decreased GI, BOP, and CAL. The treatment of RA patients with DMARDs and anti-TNF decreased the extent of CAL compared to patients without the treatment [195].

### 5.2. Effects of Treating PD on RA

The treatment of PD usually does not require pharmacological treatment, and for chronic periodontitis the mechanical debridement (scaling and root planning) of the microbial biofilm is, for the majority of cases, the treatment of choice. There is evidence supporting a somewhat limited beneficial clinical effect with the adjunct use of antibiotics [207]. Regardless, the periodontal treatment aims to reduce the microbial burden, reduce inflammation and reestablish host-microbial homeostasis.

Several studies have been performed to evaluate the influence of nonsurgical periodontal treatment (NSPT—oral hygiene instruction and mechanical disruption of microbial from tooth surfaces above and below the gingival margin) on the course of arthritis. A summary of the study methods and results obtained from each study is presented in Table 2. A recent systematic review [208] was conducted to evaluate whether NSPT in patients with both RA and PD offer benefits in terms of clinical activity and inflammatory markers of RA. A total of eight studies were included in this review and clinical (DAS28) and serological (ESR, CRP, IL-6 and TNF-α) markers were evaluated before and after treatment. The outcome of these studies supported a reduction in DAS28 and ESR scores by the periodontal treatment, while other parameters did not change after PD treatment.

Other observational clinical trials [215,216] and the systematic review with meta-analysis [220] also evaluated the effect of nonsurgical periodontal therapy on RA (and PD) biomarkers. The results of these studies demonstrated that treatment significantly reduced the levels of MMP-8, PGE2, IL-6, and t-PA in the GCF of RA patients; however, the systemic biomarkers of RA (ESR, CRP and RF) were not improved. This finding could be attributed to the use of immune-modulating medications by the RA patients (e.g., prednisolone and MTX), which would account for the low baseline DAS28 scores. However, a reduction in the DAS28 and ESR scores in RA patients after NSPT was reported in some studies [166,167,218].

The cytokine profiles in the serum of patients with PD and RA were investigated and then compared to healthy controls [221,222]. The serum levels of TNF-α were elevated in patients with combined RA/PD and were positively correlated with the RA activity and gingival BOP in patients with moderate to high disease activity. Furthermore, RA patients with increased levels of TNF-α showed increased BOP and clinical periodontal attachment loss compared to those with normal levels of TNF-α [223]. It is thus likely that the elevated levels of TNF-α contribute to periodontal inflammation in patients with RA.

Altogether, biological DMARD therapies might be suggested as an adjunctive approach for prevention or treatment of PD in patients with arthritis due to the anti-inflammatory effects of this class of medication in the periodontal tissue. On the other hand, NSPT has limited effects/benefits to reduce the RA clinical scores. However, interpretation of this evidence is restricted in the majority of studies because of important limitations, such as: Small number of studies, sample population, criteria for definition of PD and RA, the observational design of the studies, history of other medications used to treat RA that might have masked the impact of RA on the development of PD, short follow-up period, and associated environmental risk factors. For all those reasons, the findings of the current literature should be interpreted with caution, and therefore, well-controlled, randomized, prospective, multicenter large clinical trials are required to further corroborate these evidences.

## 6. Concluding Remarks

A growing body of pre-clinical animal model and epidemiological studies undoubtedly indicated that there is a strong relationship between RA and PD. The convincing biological correlation between both diseases can mainly occur by means of: (1) Genetic susceptibility (shared epitope (SE)-coding HLA-DRB1 allele), (2) microbial status (*P. gingivalis* bacteria, microbial dysbiosis at distant sites, i.e., gut microbiome, and the role of citrullination, and ACPA), and (3) inflammatory response (cytokines and pattern of immune inflammatory response toward the Th17 profile). Despite all of this, not all studies have detailed the biological mechanisms outlining how PD aggravates RA and vice-versa. In this sense, researchers should better define these mechanisms and establish cause-and-effect relationships during the progression of both conditions. This can be accomplished through well-designed pre-clinical experiments to determine systemic and tissue-specific alterations during RA and PD. Moreover, large scale, randomized, well-controlled, clinical trials are warranted to evaluate the impact of the PD treatment on the clinical course of arthritis. This should be done by means of meticulous clinical evaluation of the periodontal tissues in individuals with RA-PD, to produce a comprehensive understanding into the oral conditions of these patients. Increased knowledge of the cross-talk between both diseases could improve the clinical treatment of inflammatory diseases.

Finally, the recognition of the association between RA and PD, and the possible biological mechanisms involved during the pathogenesis of these conditions play an important role in the management of patients in need of periodontal and arthritis treatment. This means that the protocol care for patients in the treatment for RA could be altered to include periodontal examination in those patients, and in the case of a positive diagnosis of PD, the protocol treatment could be associated with the resolution of periodontal inflammation by means of NSPT. On the other hand, periodontal patients that are diagnosed with RA should have improvement of their periodontal status with the medications taken to treat arthritis, such as biological DMARDs or nonsteroidal anti-inflammatory drugs because of its immune-modulatory effects for both diseases. Taken together, rheumatologists and periodontitis should be aware of this strong association seeking to improve the treatment modalities to achieve better clinical outcomes.

## 7. Research Agenda

An increased understanding of the biological interactions and reciprocal influences between rheumatoid arthritis and periodontitis can contribute to significant advances of the study of the pathophysiology of both conditions and may also have therapeutic implications for the therapeutic management of RA and periodontitis. These conditions have various risk factors in common such as environmental, smoking, socioeconomic status, genetic (SE-coding HLA-DRB1 allele), dysbiosis of gut and oral microbiome, and inflammatory response; which suggest common pathogenic mechanisms. Moreover, there is evidence suggesting a role for periodontitis-associated bacteria (*P. gingivalis, A. actinomycetemcomitans, P. intermedia*) in citrullination. Since citrullination is characteristically increased in RA and may also participate in the pathogenesis of periodontitis, it may represent a biological mechanism bridging reciprocal influences between RA and periodontitis. Below are the key points of this review:

- Current evidence points to a pivotal role of microbiome in the pathogenesis of inflammatory conditions and its imbalance may ultimately result in the disease initiation.
- *P. gingivalis* and *A. actinomycetemcomitans* are currently the two most important microorganisms involved in the pathogenesis of PD and RA and are associated with increased citrullination. Evidence suggests that increased citrullination may also participate in tissue destruction associated with periodontitis.
- A bidirectional causal relationship between RA and PD is hypothesized, and citrullination may represent a key mechanism mediating reciprocal influences in this biological intersection.
- Clinical studies suggest that the RA treatment may ameliorate PD. Conversely, there are controversial reports on the benefits of the PD treatment in the improvement of RA. Clinical studies are difficult and limited because of a number of biases, particularly in the approach to control the influence of tobacco use. Studies in never-smokers will provide important information on the reciprocal effects of therapeutic management of RA and PD.

## Figures and Tables

**Table 1:** Evidence table summarizing the main outcomes of clinical trials evaluating the RA treatment in patients with established PD.

Study	Country	Patient Number	Objective	Study Design	Findings	Conclusions
Jung et al. (2018) [193]	Korea	64	To evaluate the adjunctive effect of DMARDs in response to NSPT in RA patients.	Prospective clinical trial. All patients received NSPT and only the RA-PD group received DMARDs. Periodontitis indices (probing depth, CAL, GI, and BOP) were evaluated at the baseline and four weeks later.	Four weeks after NSPT, the periodontal indices (probing depth reduction, and CAL gain) were significantly different in the RA group treated with DMARDs compared to the systemically healthy patients.	The study provides clinical evidence that DMARDs may have an adjunctive effect on response to NSPT in patients with RA.
Ziebolz et al. (2018) [194]	Germany	168	To investigate clinical periodontal findings in patients with RA under immunosuppressive rheumatic medications.	Cross-sectional study. Patients with RA treated with different immunosuppressive medications were involved. Periodontal parameters (probing depth, BOP and CAL) was measured.	RA medication was associated with periodontal inflammation, without differences in PD severity.	Based upon their mechanisms of action and efficacy in the reduction of systemic inflammation associated with RA-related medications, they have varying effects on periodontal inflammation.
Romero-Sanches et al. (2017) [195]	Colombia	177	To evaluate the effects of conventional drug treatment and anti-TNF therapy in patients with RA on microbiological and periodontal condition.	Prospective clinical trial. RA patients under anti-TNF therapy and under DMARD were involved. Periodontal evaluation (BOP, CAL, probing depth) and rheumatologic markers (ACPA, RF, DAS28, ESR and CRP) were measured.	The anti-TNF therapy with methotrexate resulted in lower extension of CAL. Increased ACPAs titers were associated with the presence of periodontal pathogens. BOP was associated with elevated CRP levels, and ESR was associated with a greater probing depth.	RA treatment affect the clinical condition and subgingival microbiota.
Ayravainen et al. (2017) [155]	Finland	124	To evaluate the role of antirheumatic medication in the periodontal health.	Prospective follow up clinical trial. RA patients treated with synthetic DMARD; patients with chronic RA treated with biological DMARDs. Degree of PD (probing depth, BOP and CAL) and clinical RA status (DAS28) were measured.	Periodontal status in patients with RA was worse compared to the population controls. Almost 80% of patients with synthetic DMARDs and 85% of patients with biological DMARDs suffered from PD compared to 40% of the controls.	There was no association between antirheumatic treatment and periodontal parameters.
Kobayashi et al. (2015) [196]	Japan	60	To compare the periodontal condition in patients with RA and PD before and after treatment with the anti-human IL-6 receptor (IL-6R) monoclonal antibody (Tocilizumab—TCZ) and anti-TNF therapy.	Longitudinal case control study. Patients with RA-PD treated with TCZ and patients with RA-PD who received the anti-TNF were involved. Clinical periodontal (GI, CAL, BOP and probing depth) and rheumatologic assessments (DAS28 and CRP) were assessed at the baseline and three and six months later.	Decreased levels of GI, BOP, and probing depth in patients with RA-PD after medication with anti-IL6 and anti-TNF were observed. Both therapies decreased DAS28, CRP, the number of tender and swollen joints, and serum levels of ACPA, RF, CRP, and MMP-3.	Anti-IL6 therapy significantly decreased the levels of periodontal inflammation in patients with RA-PD.
Coat et al. (2015) [197]	France	21	To evaluate the efficacy of rituximab in the periodontal parameters of patients with RA.	A cross-sectional and longitudinal study. Patients were divided in two groups: Group 1 received two doses of rituximab and group 2 received more than two courses of rituximab. The periodontal status (BOP, GI, CAL and probing depth) were measured.	Significant decrease in the probing depth and CAL were observed after six months of treatment with rituximab in group 1. Patients from group 2 presented better periodontal status than patients from group 1 before treatment with rituximab.	Anti-IL6 therapy could be beneficial to improve PD.
Kobayashi et al. (2014) [198]	Japan	20	To assess the effect of the anti-TNF inhibitor (adalimumab), on the periodontal condition of patients with RA and to compare the serum protein profiles before and after therapy.	Prospective clinical trial. Patients with RA under the adalimumab treatment were included. Periodontal indices (GI, BOP, CAL and probing depth) and rheumatologic scores (DAS28-CRP) were measured.	A significant decrease in GI, BOP, probing depth, DAS28-CRP, and serum levels of TNF-α and IL-6 after adalimumab therapy were evidenced.	These findings might suggest a promising effect of adalimumab therapy on the periodontal condition of patients with RA.
Ustun et al. (2013) [199]	Turkey	16	To evaluate the effects of host modulation with the anti-TNF therapy in periodontal tissues of patients with RA.	Longitudinal clinical trial. RA individuals were included, and periodontal indices (BOP, CAL, GI, and probing depth), GCF samples of IL-1β, IL-8 and MCP-1, and arthritis parameters (DAS28, CRP, and ESR) were measured at the baseline and 30 days after.	A decrease in the GCF volume, as well as IL-1β, IL-8, and MCP-1 levels in RA patients on the anti-TNF therapy was observed compared to the baseline. Probing depth and CAL of all patients remained unchanged. After 30 days of the anti-TNF therapy, CRP, ESR and DAS28 values were significantly lower compared to the baseline.	Host modulation might alter biochemical parameters of the periodontium in PD patients even without NSPT.
Mayer et al. (2013) [200]	Israel	58	To evaluate the effect of autoimmune diseases (AD) treated with anti-TNF on the clinical and immunologic parameters of the periodontium.	Observational clinical trial. Patients with AD were enrolled (12 RA; 12 psoriatic arthritis; 12 systemic sclerosis patients). Ten RA patients were at the anti-TNF therapy (RA+) and 12 were systemically health individuals (H). the periodontal indices (GI, BOP, CAL and probing depth) and TNF-α levels were measured.	No differences were found among the AD groups in clinical and immunologic parameters. GI was increased in the AD patients compared to the H and RA+ groups. Significantly more BOP and decreased probing depth in the SD groups were observed compared to H and RA+. Increased levels of TNF-α in the AD groups were seen compared to H and RA+.	Patients with AD diseases presented with worse PD and higher TNF-α levels than the H controls. Anti-TNF-α treatment appears to hinder this scenario.
Savioli et al. (2012) [201]	Brazil	18	To evaluate the influence and the evolution of PD in RA patients treated with anti-TNF-α.	Longitudinal and prospective clinical. RA patients on the anti-TNF treatment were included. Periodontal assessment (GI, BOP, CAL and probing depth) and rheumatologic evaluation (DAS28, ESR, and CRP) were measured at the baseline and six months later.	Eight out of 18 patients were diagnosed with PD. Periodontal indices were stable in the entire group throughout the experimental period. Significant improvement in all rheumatologic parameters were evidenced after six months of treatment with anti-TNF. This improvement was restricted to the individuals without PD.	PD patients did not improve rheumatologic parameters. Underlying PD may affect TNF blockers efficacy in patients with RA.
Mayer et al. (2009) [202]	Israel	30	To investigate the influence of the anti-TNF-α therapy on the clinical and immunologic parameters of the periodontium.	Longitudinal clinical study including 10 subjects with RA receiving anti-TNF-α; 10 RA patients without biological DMARD and 10 health control. Periodontal parameters (GI, BOP, CAL and probing depth) were measured as well as levels of TNF-α in GCF and DAS28.	The anti-TNF- α therapy decreased the GCF levels of TNF-α and lead to milder PD (decreased probing depth and CAL) compared to the RA patients who did not receive this medication. Rheumatologic markers (DAS28, CCP and RF) were similar between groups receiving or not receiving the medication.	Anti-TNF-α agents may halt periodontal inflammation and bone resorption.
Pers et al. (2008) [203]	France	40	To investigate the beneficial effects of anti-TNF-α in RA patients with coexisting PD.	Cross sectional and longitudinal study. Patients were divided into: RA patients that had already started treatment at the time of periodontal examination and RA patients that were evaluated before treatment.	A significant decrease in CAL was observed in RA patients receiving anti-TNF-α. On the other hand, increased gingival inflammation was evidenced in patients under the infliximab therapy with the coexisting PD.	Blocking the TNF-α activity may help in the treatment of PD.
Miranda et al. (2007) [204]	Brazil	34	Aiming at comparing the inflammatory activity in the GCF of RA patients and to evaluate the effect of the RA treatment on PD	Cross sectional observational study. Seventeen patients were RA positive and the other half were health control. IL-1β, -18 and the elastase activity were measured. RA patients were under NSAID medication.	Significantly lower amounts of IL-1β and elastase activity in RA individuals were observed when compared to the health control.	The NSAID treatment taken by RA patients might influence the PD status by decreasing inflammatory mediators commonly seen during the PD progression.

Disease-modifying antirheumatic drugs (DMARDs), Non-Surgical Periodontal Treatment (NSPT), Rheumatoid Arthritis (RA), Periodontal Disease (PD), Clinical Attachment Loss (CAL), Gingival Index (GI), Bleeding on Probing (BOP), Tumor Necrosis Factor (TNF), Anti-citrullinated proteins antibodies (ACPA), Rheumatoid Factor (RF), Disease Activity Score including 28-joint count (DAS28), Erythrocyte sedimentation rate (ESR), C-Reactive Protein (CRP), Interleukin (IL), Tocilizumab (TCZ), Metalloproteinase (MMP), systemically diseased (SD), Health (H), Rheumatoid Arthritis positive patients (RA+).

**Table 2:** Evidence table summarizing the main outcomes of clinical trials evaluating the effects of nonsurgical periodontal treatment (NSPT) in patients with active RA.

Study	Country	Patient Number	Objective	Study Design	Findings	Conclusions
Cosgarea et al. (2019) [209]	Romania	36	To evaluate the effect of NSPT in patients with RA and PD.	Prospective, case-controlled trial. The RA-PD group and the PD group were treated with scale and root planning (SRP). At the baseline and at three and six months after SRP the periodontal status and RA disease activity were measured.	RA patients presented a statistically significant decrease in the serum-CRP at three months. At all time-points, levels of inflammatory markers in GCF were higher in the RA-PD than in PD patients.	Only tendencies to reduction of DAS28 were observed after three and six months after NSPT.
Kaushal et al. (2019) [210]	India	40	To evaluate the effects of NSPT on the RA disease activity.	Prospective clinical trial. PD and RA parameters were examined at the baseline and eight weeks following NSPT.	Significant reduction in PI, GI, PPD, CAL and DAS28 scores were observed in patients that received NSPT compared to the untreated patients. The serum levels of ACPA, RF and CRP were not different between groups.	NSPT improved the RA disease activity and periodontal clinical conditions.
Monsarrat et al. (2019) [211]	France	22	To assess the effects of NSPT on the clinical and biochemical parameters of the RA disease activity and quality of life.	Open-label randomized clinical trial. Patients were allocated to immediate and delayed NSPT. The DAS28-ESR and health assessment questionnaire were employed. The PD and RA parameters were examined three months following NSPT.	NSPT did not lead to a significant reduction of the DAS28-ESR scores in RA patients with PD. Improvement in all periodontal parameters were evidenced after NSPT.	No improvement of the quality of life after NSPT was noted. No beneficial effect of NSPT was observed on patients with active RA.
Zhao et al. (2018) [212]	China	64	To investigate the effects of NSPT on RA.	Prospective clinical study. Patients were divided into four groups: PD patients, RA, RA-PD, and healthy controls. PD and RA parameters were examined at the baseline and one month following NSPT.	The RA-PD group had significantly higher levels of CRP, ACPA, ESR, and DAS28 than those in the RA group.	NSPT lead to improvement of rheumatologic parameters in RA-PD patients. RA showed little effect on accelerating the development of PD.
Yang et al. (2018) [213]	Taiwan	31	Aiming at investigating the effect of NSPT on the serum levels of RA-related inflammatory markers in patients with PD.	Prospective clinical trial. Patients were treated with NSPT and the serum levels of ACPA, RF, TNF-α, CRP, IL-1β, and IL-6 were measured at the end of the treatment.	NSPT significantly reduced the levels of ACPA and TNF-α in the serum of PD patients. A positive correlation was noted between the number of extracted teeth and the reduction of ACPA and IL-1β after NSPT.	RA-clinical parameter might be improved after NSPT.
Balci Yuce et al. (2017) [214]	Turkey	53	To evaluate proinflammatory cytokine and vitamin D levels in RA and PD patients before and after NSPT.	Controlled, parallel-group clinical trial. Patients were treated with NSPT and levels of vitamin D, TNF-α, OPG, and RANKL in GCF and serum were measured.	After NSPT, the levels of 25-hydroxy-vitamin D were reduced in RA-PD patients. RANKL and TNF-α levels in RA patients decreased after NSPT.	Significant improvements in clinical parameters after NSPT in both RA and PD patients were observed.
Kurgan et al. (2017) [215]	Turkey	45	To evaluate the effect of NSPT on clinical parameters and GCF levels of t-PA and PAI-2 in patients with PD, with or without RA.	Prospective clinical trial evaluating T-PA, PAI-2, CRP, DAS28, ESR and periodontal parameters were measured at the baseline and three months after NSPT.	All periodontal clinical parameters were significantly higher in the RA-PD and PD groups compared with the control group. NSPT significantly reduced the GCF t-PA levels in the RA-PD group.	NSPT significantly improves clinical periodontal parameters both in RA-PD and in the PD patients.
Kurgan et al. (2016) [216]	Turkey	66	To evaluate whether NSPT influences the levels of MMP-8, IL-6 and PGE2 in the GCF, and serum levels of RA biomarkers in patients with RA-PD.	Observational clinical trial. Patients were evaluated at the baseline and after three months of NSPT.	The GCF levels of MMP-8, PGE2 and IL-6 were higher in all groups than the control. After NSPT, there were significant decreases in the GCF levels of MMP-8, PGE2 and IL-6 from patients with RA-PD.	NSPT may provide beneficial effects on local inflammatory mediators via decreases in the GCF of inflammatory biomarkers.
Bıyıkoglu et al. (2013) [217]	Turkey	30	To evaluate clinical and biochemical outcomes of NSPT on the serum and GCF in PD patients with or without RA.	Single-centered interventional study. Clinical and biochemical periodontal (IL-1β, and TNF-α) and RA (DAS28) parameters were obtained at the baseline, one, three, and six months after NSPT.	The DAS28 decreased significantly after NSPT in the RA-PD group. The serum TNF-α of the PD group were significantly higher than those of RA-PD. After NSPT, no changes were noted in the levels of these cytokines. The GCF of the IL-1β levels decreased in both groups after NSPT. At six-months, the GCF of the IL-1β levels were significantly lower than the baseline.	NSPT might be beneficial in decreasing local inflammatory markers of PD and RA.
Okada et al. (2013) [218]	Japan	55	To evaluate whether NSPT affect the serum antibodies to *P. gingivalis* and citrulline levels in relation to the disease activity of RA.	Interventional and prospective clinical trial. Periodontal and rheumatologic parameters and serum levels of cytokine and inflammatory markers citrulline and IgG to *P. gingivalis* were examined at the baseline and eight weeks later.	The NSPT group exhibited a significantly greater decrease in DAS28-CRP, the serum levels of IgG to *P. gingivalis*, and citrulline than the control group. The serum levels of IgG to *P. gingivalis* were positively correlated with those of the ACPA antibodies.	The findings suggest that NSPT decreases DAS28-CRP and the serum levels of IgG to *P. gingivalis* and citrulline in the RA patients and may reflect a role of *P. gingivalis* in the protein citrullination.
Erciyas et al. (2013) [165]	Turkey	60	Aiming at evaluating the effects of NSPT on clinical periodontal measurements and systemic inflammatory mediator levels in RA-PD patients.	Observational prospective cohort study. Thirty patients were RA-PD with a moderate to high DAS28 score and the others were RA-PD with a low DAS28 score. The ESR, CRP, TNF-α levels in serum, DAS28 and periodontal parameters were evaluated at the baseline and after three months of NSPT.	The ESR, CRP, TNF-α levels in serum, DAS28 and periodontal parameters exhibited similar and significant reduction three months after the NSPT.	These findings might indicate beneficial effects of NSPT in reducing RA severity as measured by a significant decrease in inflammatory markers in the serum and DAS28 score in low or moderate to highly active RA patients with PD.
Pinho et al. (2009) [219]	Brazil	75	To evaluate the effects of NSPT on clinical and laboratory parameters in patients with RA and PD.	Clinical and interventional trial. Patients were assigned to five groups according to the presence or absence of RA and PD and with or without NSPT. Clinical periodontal indices, DAS28, CRP, ESR and alpha-1 acid glycoprotein (AAG) were measured at the baseline, three and six months after NSPT.	Significant reduction of periodontal clinical parameters was observed in three and six months after NSPT. A significant decrease in the DAS28 scores of patients with RA-PD were observed when compared to the RA patients that underwent NSPT at the baseline and after three months, but no differences were found after six months.	NSPT might be considered an adjunctive approach to reduce the levels of DAS28 in the RA patients. No other parameters for RA (CRP, ESR and AAG) were significantly affected by NSPT.
Ortiz et al. (2009) [166]	USA	40	To investigate the effect of NSPT on the signs and symptoms of RA in patients treated with or without anti-TNF-α.	Clinical and interventional trial. RA-PD patients under the RA treatment were enrolled. Half of them received NSPT and the other half did not. Clinical periodontal parameters and RA disease activity levels (DAS28 and ESR) were measured at the baseline and six weeks later.	NSPT lead to a significant decrease in the mean DAS28, ESR, and serum TNF-α levels. No significant decrease in these parameters were observed in the untreated control patients. The anti-TNF-α therapy decreased the clinical signs of periodontitis characterized by a reduction in CAL, BOP, probing depth and GI.	The control of PD and inflammation by means of NSPT might contribute to a reduction in the signs and symptoms of active RA.
Al Katma et al. (2007) [167]	USA	29	To evaluate the impact of NSPT on the activity of RA.	Prospective clinical trial. Seventeen RA-PD patients received NSPT and 12 did not. Patients were under the DMARD medication. RA measurements (DAS28 and ESR) and PD indices (CAL, probing depth, BOP, GI) were measured at the baseline and eight weeks after.	Significant decrease in the DAS28 and ESR levels were observed in patients under the NSPT treatment compared to the untreated control. NSPT led to a significant improvement in all periodontal clinical parameters including PI, GI, BOP, and probing depth.	NSPT might reduce the severity of periodontal patients with active RA.

Scale and root planning (SRP), Non-Surgical Periodontal Treatment (NSPT), Rheumatoid Arthritis (RA), Periodontal Disease (PD), Clinical Attachment Loss (CAL), Gingival Index (GI), Bleeding on Probing (BOP), Gingival Crevicular Fluid (GCF), Tumor Necrosis Factor (TNF), Anti-citrullinated proteins antibodies (ACPA), Rheumatoid Factor (RF), Disease Activity Score including 28-joint count (DAS28), Erythrocyte sedimentation rate (ESR), C-Reactive Protein (CRP), Interleukin (IL), Metalloproteinase (MMP), Osteoprotegerin (OPG), Receptor Activator of the Factor Nuclear Kappa B Ligand (RANKK-L), alpha-1 acid glycoprotein (AAG), Immunoglobulin G (IgG).

## Abbreviations

ABL	Alveolar bone level
ACPA	Anti-citrullinated proteins antibodies
AIA	Antigen-induced arthritis
BOP	Bleeding on probing
BSA	Bovine serum albumin
CAL	Clinical attachment level
CIA	Collagen-induced arthritis
CRP	C-reactive protein
CT	Computer tomography
DAS28	Disease Activity Score including 28-joint count
DMARD	Disease-modifying antirheumatic drugs
ESR	Erythrocyte sedimentation rate
EULAR	European league against rheumatism
GC	Glucocorticoids
GCF	Gingival crevicular fluid
GI	Gingival index
GM-CSF	Granulocyte macrophage colony-stimulating factor
IgG	Immunoglobulin G
IL-1β	Interleukin-1β
IL-17	Interluekin-17
IL-6	Interluekin-6
LtxA	Leukotoxin A
MAMP	Microbe-associated molecular pattern
MMP	Matrix metalloproteinase
MRI	Magnetic resonance imaging
NHANES	National Health and Nutrition Examination Survey
NHS	National Health Service
NSAID	Non-steroidal anti-inflammatory drugs
NSPT	Non-surgical periodontal treatment
PAD	Peptidylarginine deiminase
PAMP	Pathogen-associated molecular pattern
PD	Periodontal disease
PGE2	Prostaglandin E2
PPD	Probing pocket depth
RA	Rheumatoid arthritis
RANK-L	Receptor activator of NF-κB ligand
RF	Rheumatoid factor
ROS	Reactive oxygen species
SE	Shared-epitope
TGF-β	Transforming growth factor-β
TNF-α	Tumor necrosis factor-α
VAS	Visual analogue scale
WT	Wild-type
